# Blu-ray-sensitive localized surface plasmon resonance for high-density optical memory

**DOI:** 10.1038/srep36701

**Published:** 2016-11-07

**Authors:** Shencheng Fu, Xintong Zhang, Qiang Han, Shuangyan Liu, Xiuxiu Han, Yichun Liu

**Affiliations:** 1 Center for Advanced Optoelectronic Functional Material Research, Northeast Normal University, and Key Laboratory of UV-Emitting Materials and Technology (Northeast Normal University), Ministry of Education, Changchun 130024, P. R. China

## Abstract

Tunable spectrum-response is desired for efficient photo-energy transformation. Blu-ray (~405 nm) and polarization sensitive Ag/TiO_2_ nanocomposite films are thus fascinating in application of fast-response and high-density optical memory device. The Ag/TiO_2_ film has the ability of replicating hologram based on optical coherence by laser-stimulated dissolution of Ag nanoparticles (NPs). The rate and efficiency of the dissolution are supposed to be enhanced by introducing uniform and small-sized Ag NPs in TiO_2_ nanoporous films. However, no effective methods have been proposed to resolve this issue by now. Here, we develop a simple method of thermal-reduction to obtain high-density, space-dispersed and extremely small-sized Ag NPs in TiO_2_ nanoporous films pretreated with tannic acid. The film shows both high and narrow absorbance band centered at ~405 nm. Diffraction efficiency of the blu-ray holographic storage in the Ag/TiO_2_ film is improved by one order of magnitude compared to the traditional UV-reduced sample. Based on such properties, polarization-multiplexing holograms are able to be written at 405 nm and readout with little crosstalk. This work provides effective solutions for sensitizing localized surface plasmon resonance at near-UV region, extending the growth range of Ag NPs in the volume of TiO_2_, and resultantly, realizing high-density optical memory.

Big Data desires massive information storage with the characteristics of safety, energy-saving and long-life^1^. Compared to DVD, *Blu-ray Disc* (BD) adopts a blue-violet laser at ~405 nm as writing source, which has shorter wavelength and higher numerical aperture to form a smaller light spot and pack information more densely^2^,^3^. Based on a similar optical system to BD storage, hologram with multiplexing technique can replace traditional information-bit via a series of coherent laser irradiations, which will promote the ability of large-volume optical storage beyond TB level^4^,^5^,^6^,^7^. A holographic storage medium, sensitive at the blue-violet region, is thus supposed to be the key component in the progress of mass information storage.

Noble metal nanoparticles (NPs) coupled with polarized light induces their conduction electrons to oscillate collectively with a resonant frequency, i.e. localized surface plasmon resonance (LSPR) which is closely related to the electron density, particle size and shape, refractive index of the surrounding medium, and inter-particle spacing^8^,^9^,^10^,^11^,^12^,^13^. It is feasible to tune LSPR to blue-violet region by fabricating small-sized NPs. Recently, (plasmonic-metal)/semiconductor hybrid nanostructures have received extensive attention for their variable optical response, resulting in the application in photocatalysis^14^, surface-enhanced spectroscopy^15^, sensor^16^, solar cells^17^ and information storage^18^. As a promising holographic memory medium, titanium dioxide nanoporous films loaded with Ag NPs can produce charge-separation in a certain direction under the polarized resonant irradiation, and storing hologram with a high spatial resolution at nano-scale^19^. TiO_2_ plays a key role as electron-transferring bridge to realize the transformation from Ag to Ag_2_O^13^. The written hologram thus consists of the modulation of absorbance and refractive index of Ag/Ag_2_O^18^, as well as periodic morphology change of Ag NPs^19^,^20^.

However, Ag NPs tends to gather with each other under the traditional fabrication of electronic-deposition^12^ or UV-reduction^21^, resulting in variousness of Ag NP size and broad absorption band of LSPR from visible to near infrared region. Mesoporous titania films was proved to be an excellent template for the controlled growth of small-sized Ag NPs^22^,^23^,^24^, followed by thermal reduction in a Ag(NH_3_)_2_^+^ solution at 200 °C^25^. Whereas, it is still difficult for Ag/TiO_2_ films to get both high value and narrow bandwidth of absorbance at ~405 nm. Recently, an effective optical manipulation of inserting an additional excitation with lower-frequency and orthogonal polarization-state in the blu-ray holographic recording was proved to be able to tailor Ag NP size and enhance photoelectron-transformation efficiency at blue-violet wavelength^26^. However, the original issue to obtain high-density and blu-ray sensitive Ag NPs in TiO_2_ nanoporous films has not been resolved yet.

Here, we report that uniform and small-sized Ag NPs can be deposited in TiO_2_ nanoporous films with a simple thermal reduction by pre-absorbed tannic acid. The fabricated storage medium presents a narrow absorption band centered at ~405 nm resulting in the blu-ray holographic recording with high-efficiency. Polarization-multiplexed holograms are thus written by the blue-violet laser in the Ag/TiO_2_ film with high efficiency, and readout with little crosstalk.

## Methods

### Sample preparation

**Figure 1:**
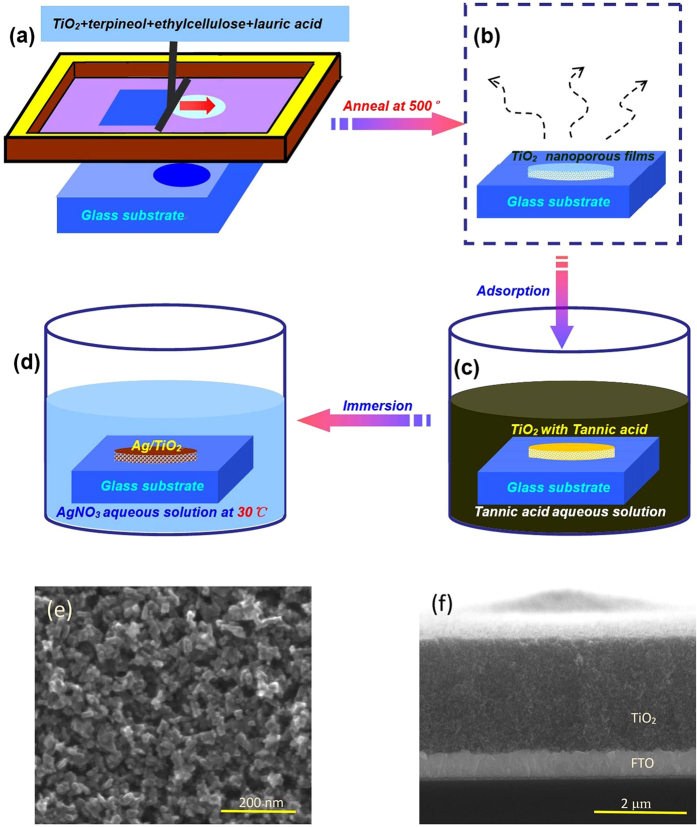
Fabrication process of small-sized Ag/TiO_2_ nanocomposite films. **(a)** TiO_2_ nanoporous films prepared on glass slides by a screen-printing method. **(b)** Heat treatment to remove the polymer. **(c)** Adsorption of TA on the surface of TiO_2_ **(d)** Thermal reduction of Ag nanoparticles in TiO_2_ nanoporous films. **(e)** Top-view and **(f)** cross-section of SEM images of porous TiO_2_ films obtained by screen-printing on the substrate of FTO.

TiO_2_ nanoporous films were prepared on glass slides by a screen-printing method from the nominal composition of TiO_2_/terpineol/ethylcellulose/lauric-acid of 1/6/0.3/0.1, followed by heating at 135  °C for 10 minutes on the hot plate, and then annealing at 500  °C for 2 hours to remove the polymer, as shown in Fig. 1(a,b). The porous morphology of the TiO_2_ surface was shown in Fig. 1(e). The average pore size is in the range 25–50 nm, which is larger than that of the titania film via sol-gel process and dip-coating^22^,^23^,^24^,^25^ (see Fig. S1 in the Supporting Information). The typical thickness of the oxide semiconductor film after one operation is about 2.4 μm, measured by cross-sectional SEM, as shown in Fig. 1(f). For the advantage of film thickness, both large-population and small-sized Ag NPs was proved to be formed in screen-printed titania film than the dip-coated one (see Fig. S2 in the Supporting Information). Subsequently, tannic acid (TA) was chosen as an electron donor in reduction of silver ions. Potassium carbonate solution with the concentration of 0.009 mmol/L was dipped into the TA solution of 39 mL with the concentration of 0.002 mmol/L to fabricate a mixed solution with the PH value of 8.5, measured by a PH meter. The TiO_2_ nanoporous film was immersed in the mixed solution for 3 h in order that TA molecules can be adsorbed on the surface of TiO_2_ sufficiently (Fig. 1c). The nitrogen-dried TiO_2_ nanoporous film with TA molecules was again immersed in the solution of 0.01 M silver nitrate (AgNO_3_) (100 mL) mixed with ethanol (2 mL) by water-curing treatment at 30 °C for different times (Fig. 1d). Ag NPs were deposited in the TiO_2_ nanoporous films high-efficiently by thermal-catalytic reduction of Ag^+^ ions. During the process of water-curing treatment, the sample color turned to brownish-gray gradually due to LSPR absorption of the deposited Ag NPs. Subsequently, the absorption spectra of Ag NPs of different water-curing times were measured with an UV-Vis spectrophotometer. The environmental temperature is ~300K and the relative humidity is 40%.

For the UV-reduction method, the TiO_2_ nanoporous film was immersed in the AgNO_3_ solution and irradiated for 20 min in air at room temperature with ultraviolet light, of which the power density irradiating at the surface of the sample is ∼1.7  mW/cm^2^, rinsed with pure water, and finally irradiated again with ultraviolet light for 5 min to reduce resident Ag^+^ ions. Ag NPs were deposited in the TiO_2_ nanoporous film by photocatalytic reduction of Ag^+^ ions.

### Optical setup for holographic reconstruction

**Figure 2:**
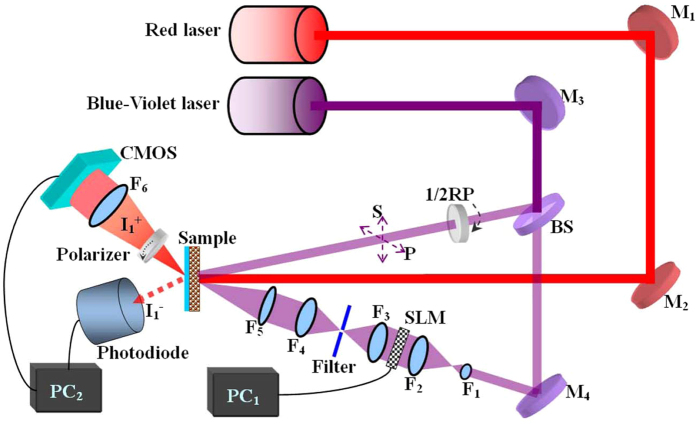
Experimental configurations for polarization multiplexed holographic storage and multiplexed hologram reconstruction in Ag/TiO_2_ nanocomposite films: M, mirror; BS, beam splitter; RP, retardation plate; F, lens.

The simplified scheme of the experimental setup is shown in Fig. 2. The diffraction grating was recorded with two linearly polarized laser beams from *BlueMode* lasers (Topical Phontics, 403.4 nm). The angle between the writing beams was fixed at 14.5°, resulting in a grating period of Λ = 1.598 μm, according to Λ = *λ*/2sin(*θ*/2), where *λ* is the wavelength of the writing beam. The intensities of the interfering beams were the same and equal to 10 mW. A half-wave plate was used to adjust the electric-field vector of oscillating in the plane of incidence or perpendicular to it, i.e., *p*- or *s*-polarization. One of the writing beams was expanded by a beam expander after spatial filter, collimated to pass through a spatial light modulator (SLM), and then focused onto the center of the Ag/TiO_2_ film. The other beam was superimposed on the same spot as a reference beam. A red laser (Changchun New industries Optoelectronics Tech. Co. Ltd.) generating 671 nm s-polarized light was used as a probe source to monitor the reconstructed holographic image which can be collected by a CMOS video camera. The power of the 671 nm laser was set as 0.5 mW to minimize the destructive effect of readout radiation which can in principle also lead to photochemical interactions. Then the hologram build-up was observed by Confocal Laser Scanning Microscope (CLSM, FluoView FV1000, Olympus, Tokyo, Japan). After removing SLM, the first-order diffraction signal was registered on a photodiode interfaced with a computer. Diffraction efficiency of holographic gratings, taking *Fresnel losses* into account, can be calculated accordingly, which is defined as the ratio between intensities of the first-order diffracted beam and the incident beam after passing through the sample.

## Results and Discussion

**Figure 3:**
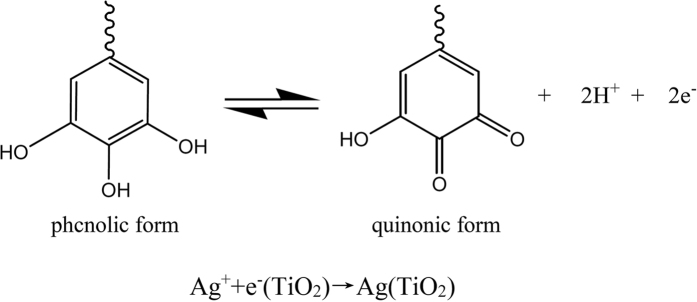
Chemical structure for phcnolic and quinonic forms of tannic acid, and the subsequent reduction reaction of silver nanoparticles.

Tannic acid has weak acidity due to the phenolic hydroxyl groups in the structure, and can adsorb to TiO_2_ surface chemically in form of monolayer. After heating, the space-dispersed TA molecules release sufficient electrons for silver ions, resulting in the *in-situ* formation of small-sized Ag NPs in the nanoporous TiO_2_ films. The chemical reactions are shown in Fig. 3.

### Photochromism

**Figure 4:**
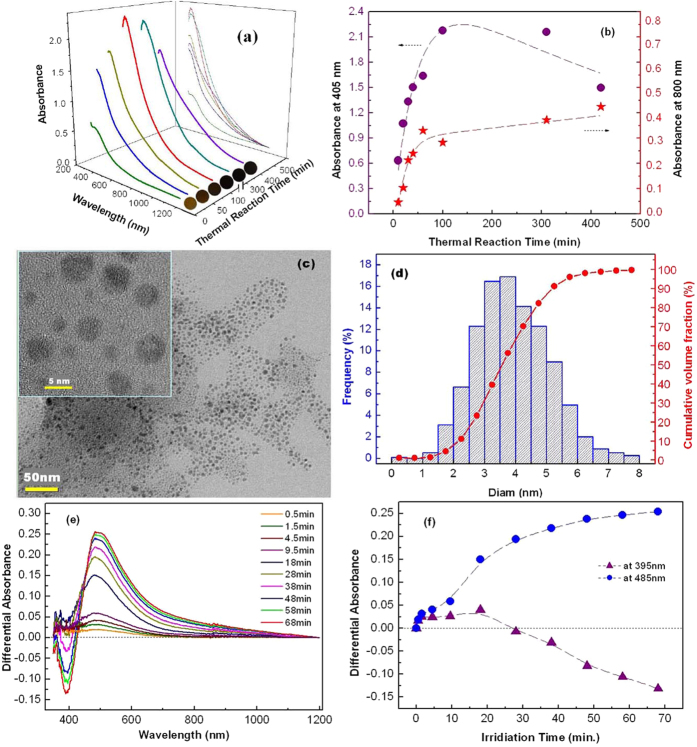
**(a)** Absorption spectra of the Ag/TiO_2_ film for different thermal-reaction periods. **(b)** Absorbance at 405 nm and 800 nm versus thermal-reaction time. **(c)** TEM photographs of Ag NPs in the nano-composite film after water-curing treatment at 30 °C for 100 min. TiO_2_ NPs in the films were dissolved with HF solution to remove interference. High resolution image is inserted. **(d)** The size distribution histograms and cumulative percentage of volume fraction of Ag NPs derived from TEM photographs. **(e)** Differential absorption spectra of the Ag/TiO_2_ film irradiated with linearly polarized blu-ray (403.4 nm, 0.5  mW) for different periods. **(f)** Time-courses of the hole-burning centered at 395 nm and the absorption accumulation at 485 nm. The lines are guides for the eyes.

A series of absorption bands of the TA-reduced Ag/TiO_2_ film as a function of thermal reaction time are shown in Fig. 4(a), covering UV, visible and near-inferred (NIR) regions (350 nm-1200 nm). The color of the sample presents from pale yellow to light brown and finally to dark brown versus immersion time. Absorbance at ~405 nm is enhanced dramatically when increasing the reduction time less than 100 min. Whereas, further thermal reaction induces an obvious decrease at the blu-ray wavelength and a slight increase at 800 nm, as shown in Fig. 4(b). Commonly, larger-sized spheral Ag NPs resonate with the light at longer wavelength, as evidenced by AFM observation^20^. The accumulated absorbance in NIR region indicates that the smaller-sized Ag NPs tend to gather with each other after saturation of NP population. Thus thermal reduction of silver ions by TA in the TiO_2_ film for 100 min may be the optimized condition for obtaining a large population of small-sized Ag NPs. Figure 4(c) shows the spherical morphology of the Ag NPs, observed by transmission electron microscopy (TEM). Cumulative volume fraction (Fig. 4d) shows the small Ag NPs (<5 nm) occupied a considerable volume fraction of ~90%. The large and ellipsoidal Ag NPs (over 8 nm) is almost nonexistent. The Ag NPs by TA-aided reduction present much more uniform size-distribution than that obtained by UV-reduction (see Fig. S3 in the Supplementary Information). According to *Lambert-Beer Law*, the photo-energy from UV source decreases exponentially along the sample depth during UV-reduction, as followed:


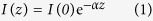


where *I* is the transmitted light intensity, *z* is the position of the sample depth and *α* is absorption coefficient. The inhomogeneous distribution (see Fig. S4 in the Supplementary Information) and scattering of UV light in TiO_2_ nanoporous films result in the random nucleation sites, which may be too close to each other. Thus the aggregation and growth of small-sized Ag NPs occur easily. While tannic acid molecules, absorbed on TiO_2_, provides more spatial-dispersed reducing sites, inhibiting Ag NPs gathering effectively.

The high-density and small-sized Ag NPs provide possibility for efficient blu-ray recording. Differential absorption spectra of the Ag/TiO_2_ film for different irradiation times were obtained by linearly polarized blu-ray excitation from the blue-violet laser of 0.5 mW, as shown in Fig. 4(e). At the beginning stage of several minutes, a broad-range increase of absorbance from 400 nm to 750 nm is produced. Then further irradiation results in both an obvious hole-burning of absorbance centered at 400 nm which behaves deeper with increasing the exposure time, and the proceeded accumulation centered at 485 nm (Fig. 4f). In fact, part of photo-excited electrons may recombine with the Ag^+^ ions in the adsorbed water at the surface of TiO_2_ or nonresonant Ag NPs^27^. Thus the re-deposition and growth of Ag NPs in the porous TiO_2_ film contribute to the absorbance accumulation at the longer wavelength.

### Holographic Dynamics

**Figure 5:**
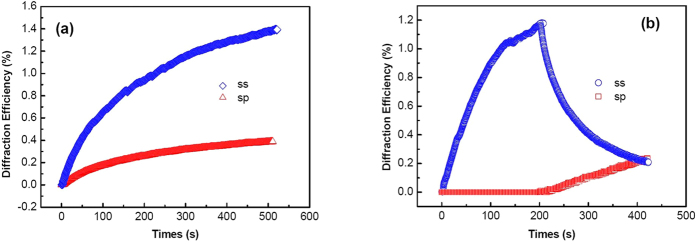
**(a)** Time dependence of the first-order diffraction efficiency in (*ss*) and (*sp*) recording configurations in the Ag/TiO_2_ nanocomposite film; and **(b)** Competition growth of the two holographic gratings: the (*ss*) grating recording separately in the first 200 s, followed by the overlapping with the (*sp*) grating recorded from 200 s to 400 s.

The apparent hole-burning at ~400 nm play a key role in the formation of the blu-ray hologram in the Ag/TiO_2_ film. Here, the s-polarized 671 nm laser beam was chosen to monitor the dynamics of the holographic grating inscribed by two coherent parallel (*ss*) or orthogonal (*sp*) polarized blue-violet beams (403.4 nm), as shown in Fig. 5(a). The kinetic behaviors of diffraction efficiency for the two kinds of holographic gratings both present standard exponential growth. The maximum values of diffraction efficiency of 1.4% and 0.3% appear at ~500 s, for the (*ss*) and (*sp*) gratings, respectively. The blu-ray storage efficiency for the Ag/TiO_2_ film is improved by one order of magnitude compared to that of the UV-reduced sample (see Fig. S5 in the Supplementary Information). It was proved that the photo-dissolution of Ag NPs in TiO_2_ nanoporous films is dependent on the polarization state of excitation light^19^,^25^. Hence, blu-ray holographic recording with polarization multiplexing can be realized in the Ag/TiO_2_ film. The light diffracted from the (sp) grating in the Ag/TiO_2_ film has a strong orthogonal component of polarization state to the zero-order one, measured by a polarimeter (Thorlabs PAX5710), while the diffracted light remains the original polarization state when the readout light passes through the (*ss*) grating in the Ag/TiO_2_ film. Thus the polarization-multiplexed holographic dynamics can be monitored by collecting *s*- and *p*-polarized signals at +1 order and −1 order diffracted position, respectively, into the polarizer-filtered photodiodes. As shown in Fig. 5(b), in the first 200 s, only the (*ss*) grating was recorded. After that, one of the writing beam was changed from *s*- to *p*-polarization state by a half-wave plate of 403.4 nm, overlapping the original excited region with the (*sp*) grating recording. The *p*-polarized diffraction intensity presents gradual increase, paid by an exponential decrease of *s*-polarized signal synchronously. At 400 s, the two polarized diffraction intensities reach the same value.

**Figure 6:**
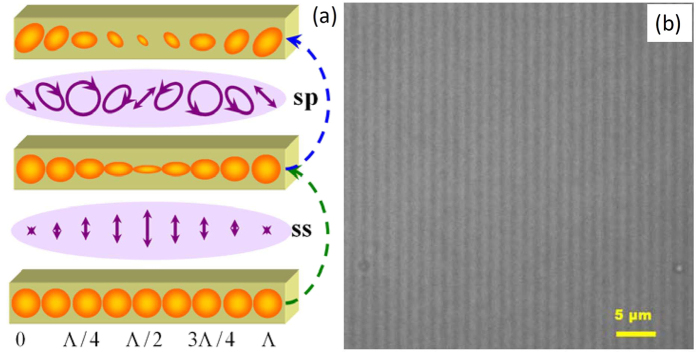
**(a)**Sketch of peridic distributions of Ag NPs after (*ss*) and (*sp*) recordings. **(b)** Holographic-grating microphotograph, Λ = 1.598 μm.

The whole holographic dynamics can be explained by anisotropic-photo-dissolution and reshape by the linearly or circularly polarized irradiation at 403.4 nm, as shown in Fig. 6(a). For (*ss*) polarization configuration, the resulting electric field of the optical waves oscillates perpendicularly to the grating wave-vector. The morphology of Ag NPs at different sites is modified differently. The Ag NPs around the center of the bright region of the interference fringes (*x* = Λ/2) is modified to the greatest extent by the polarized light while the shape of the Ag NPs around the center of the dark region of the interference fringes (*x *= 0) changes little. In the mutually orthogonal linear polarization, (*sp*) geometry, the intensity of the light is constant along the grating wave-vector. Whereas, as the resultant polarization states for (*sp*) configuration are varying between linear, elliptical, and circular ones, the shape change of the Ag NPs is still modulated periodically along the direction of grating vector. The periodical consumed Ag NPs are thus further modified perpendicular to the electrical vector of light field of (*sp*) interference, resulting in periodical variation of both the mass-density and orientation of Ag NPs. The holographic gratings in the Ag/TiO_2_ film were observed by CLSM accordingly after holographic recording for ~400 s. Then a 20-mW 488 nm laser (Cyan OEM, Spectra Physics) was chosen as the exciting beam. Interference fringes with the period of ~1.6 μm were observed after mixing the (*ss*) grating with the (*sp*) one, as shown in Fig. 6(b).

### Multiplexed Hologram Reconstruction

**Figure 7:**
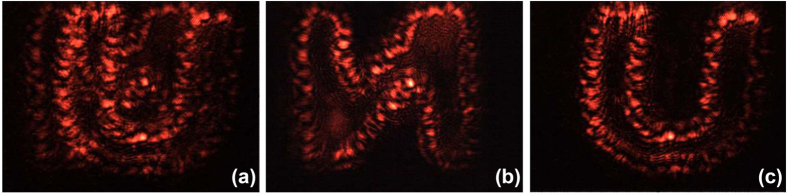
Reconstruction of the stored image with different polarization states. **(a)** No polarizer added; **(b)** Reconstructed image after setting the diaphanous direction of the polarizer paralleled to *s*- polarization state; and **(c)** to *p*- polarization state.

The efficient recording and distinguishing of the polarization-multiplexed holographic grating provide possibility for the mixed hologram storage at one spot in the Ag/TiO_2_ film. An *s*-polarized 671 nm beam which play little role in erasing the stored holograms was used to reconstruct the multiplexed holograms. A similar optical-operation to the holographic grating recording as mentioned above was carried out. Two coherent *s*-polarized blu-ray beams, one of which was loaded with object information of a letter “N”, were converged at one spot in the Ag/TiO_2_ film for ~200 s. And then the reference beam was changed from s- to *p*-polarization state, loading a letter “U” to the object beam simultaneously. The subsequent operation also continued for ~200 s to modulate the multiplexed images with the same brightness. Then the two reconstructed holograms appear simultaneously, as shown in Fig. 7(a). Subsequently, a polarizer were added in the diffracted beam and set its diaphanous direction paralleled to *s*- or *p*-polarization state, resulting in the separate appearance of holographic images. As shown in Fig. 7(b,c), the polarization multiplexed images were distinguished easily.

The strategy for synthesis of small-sized Ag NPs in TiO_2_ nanoporous films presents an excellent performance of polarization-sensitive optical-memory in near-UV region. It is possible to obtain high-efficiency reduction of Ag NPs in a thick semiconductor film as TA molecules are absorbed at any site of TiO_2_ nanoporous films, which can be applied to volume holographic storage of plasmon. Along this route, high-performance holographic memory of Ag/TiO_2_ films with abilities of distinguishing wavelength, polarization and spatial information, is expected to be realized. The noble mental particles with small size may also serve as excellent media for the nano-storage and integrate well with other photonic devices in all-optical computer.

## Conclusions

The Ag/TiO_2_ nanocomposite film with high sensitivity to blu-ray (~405 nm) was fabricated by a simple thermal reduction of silver ions by tannic acid. The obtained storage medium contains the Ag NPs with the mean diameter of only 4 nm, and presents a narrow LSPR absorption band centered at ~405 nm. The polarization-dependent LSPR results in the anisotropic photo-dissolution of Ag NPs when interfaced with TiO_2_. Based on such properties, the film was well applied to polarization-multiplexed holographic storage. Periodical variation of the mass-density and orientation of Ag NPs play a key role in the no-crosstalk holographic reconstruction. As the scale of TA adsorption on TiO_2_ is not limited to the surface of the nanoporous film but can be extended to its whole volume region, the multi-dimensional holographic storage in the wavelength, polarization and spatial orthogonal vectors for Ag/TiO_2_ films may be developed along the thermal-reduction synthesis route.

## Additional Information

**How to cite this article**: Fu, S. *et al.* Blu-ray-sensitive localized surface plasmon resonance for high-density optical memory. *Sci. Rep.* **6**, 36701; doi: 10.1038/srep36701 (2016).

**Publisher’s note**: Springer Nature remains neutral with regard to jurisdictional claims in published maps and institutional affiliations.

## Supplementary Material

Supplementary Information
