# Supplementary material for: Blu-ray-sensitive localized surface plasmon resonance for high-density optical memory
Source: Sci Rep. 2016 Nov 7;6:36701. doi: 10.1038/srep36701 (PMC5098189; doi:10.1038/srep36701)
Supplement: Supplementary Information [file srep36701-s1.doc]

Blu-ray-sensitive localized surface plasmon resonance for high-density optical memory

Shencheng Fu^1^, Xintong Zhang^1,*^, Qiang Han^1^, Shuangyan Liu^1^, Xiuxiu Han^1^ & Yichun Liu^1,*^

^1^Center for Advanced Optoelectronic Functional Material Research, Northeast Normal University; and Key Laboratory of UV-Emitting Materials and Technology (Northeast Normal University), Ministry of Education, Changchun 130024, P. R. China. ^*^Correspondence and requests for materials should be addressed to X. Zhang (email: [xtzhang@nenu.edu.cn](mailto:xtzhang@nenu.edu.cn)) and Y.Liu ([ycliu@nenu.edu.cn](mailto:ycliu@nenu.edu.cn)). Tel./Fax.: +86 43185099772.


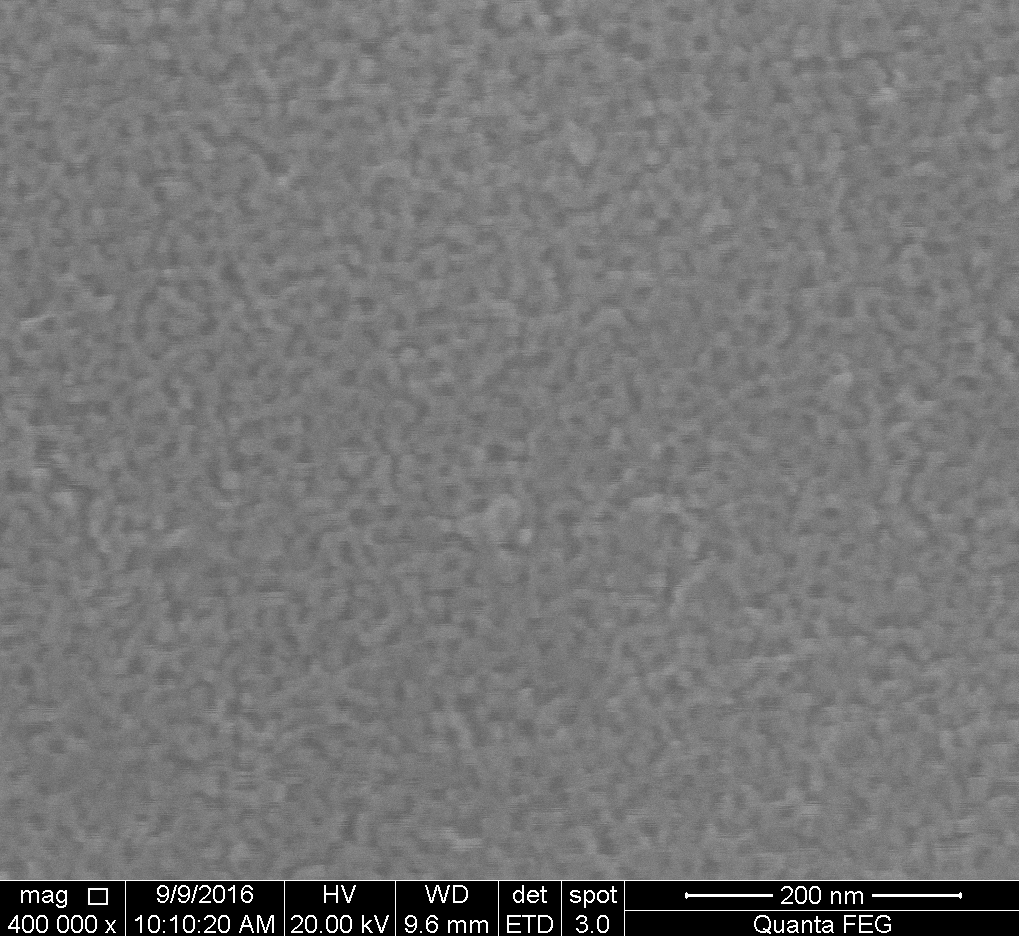

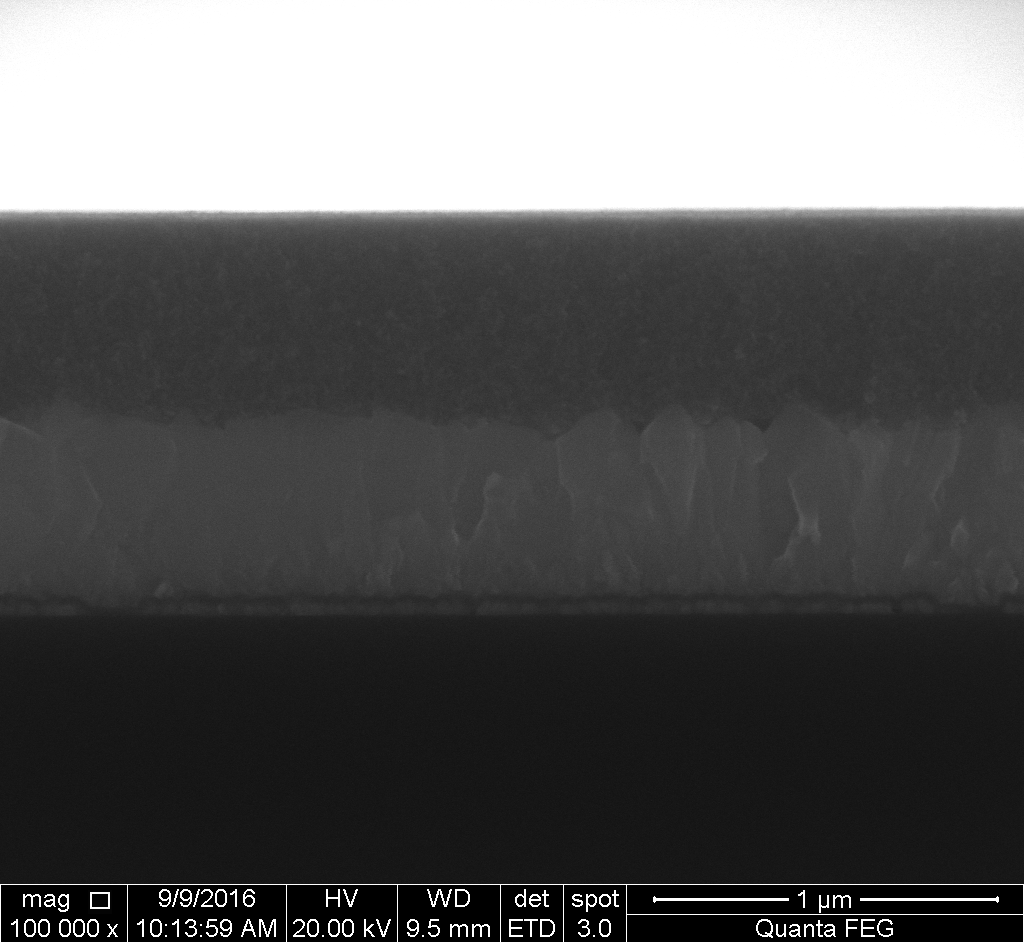


(a)

200 nm

1 μm

FTO

TiO_2_


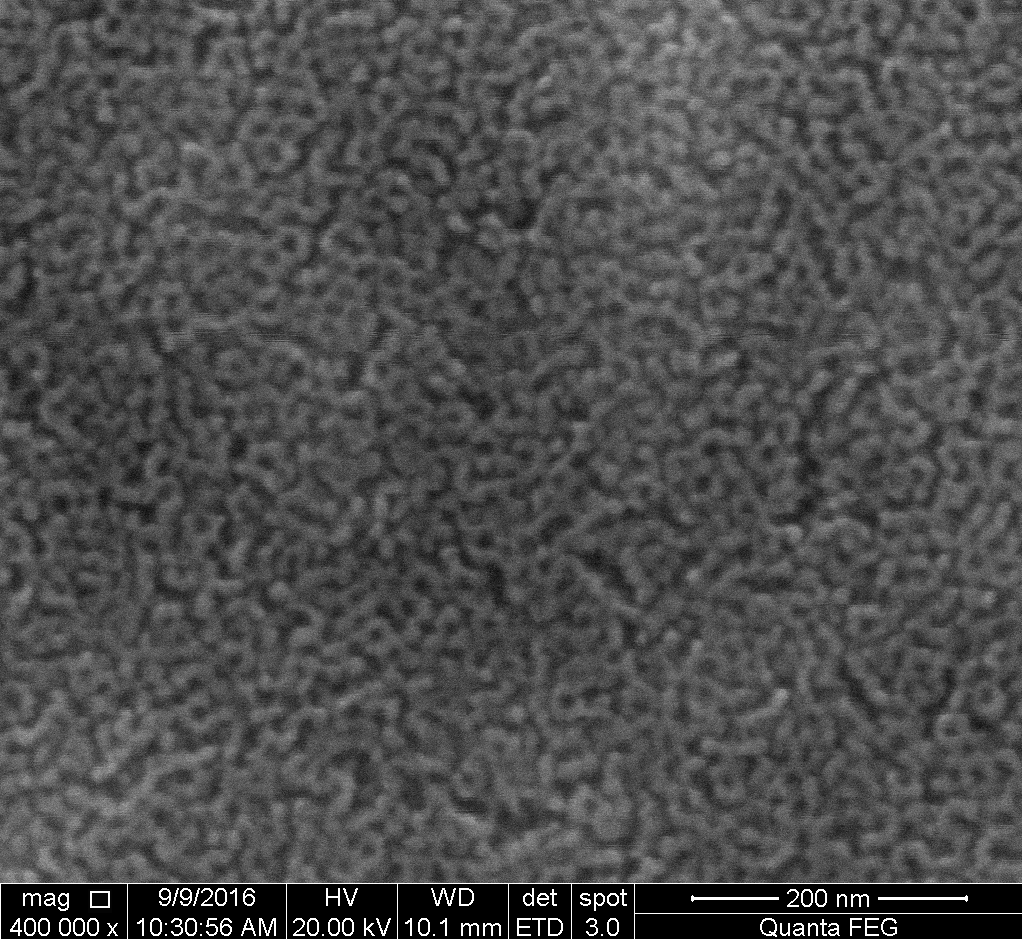

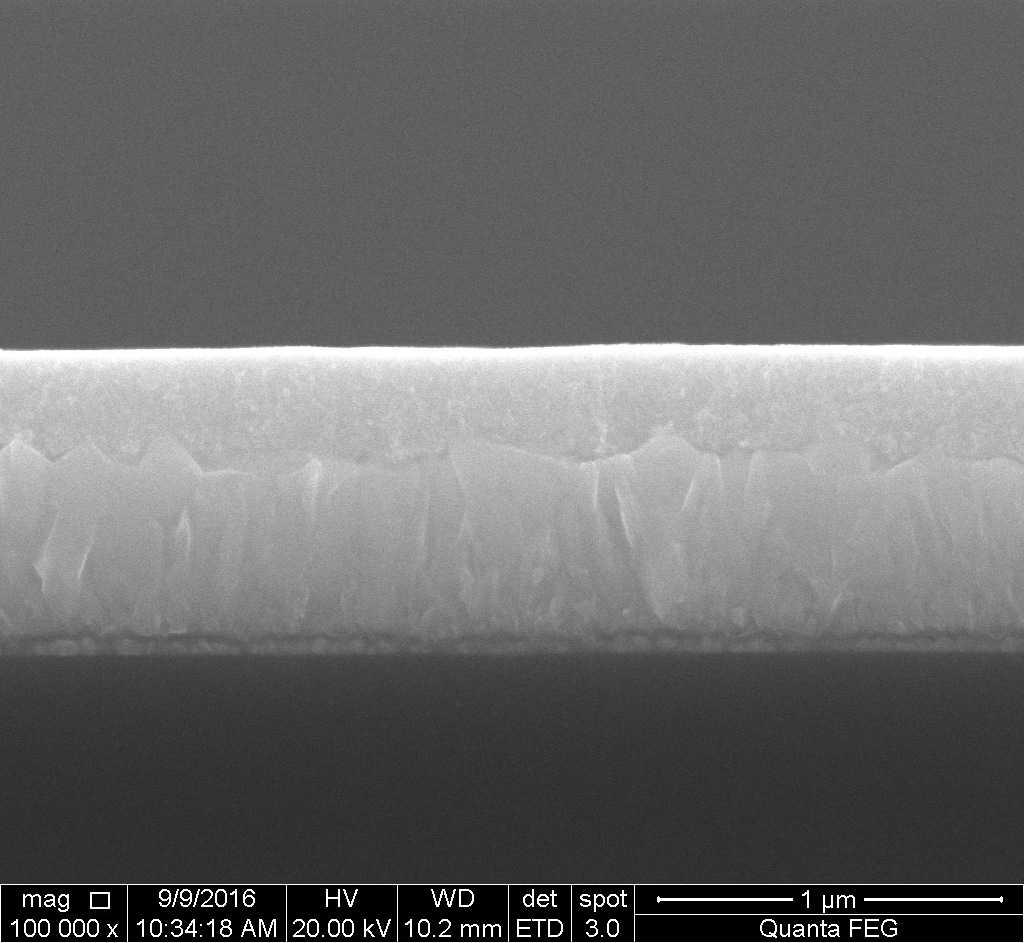


(b)

200 nm

TiO_2_

FTO

1 μm

Figure S1 Top-view and cross-section of SEM images of porous TiO_2_ films obtained by dip-coating as **(a)** *Ref. 15* (*Appl. Opt. 2012,51, 3357*) and **(b)** *Ref. 19* (*Adv. Mater. 2010, 22, 3166*)

Figure S2 Absorption spectra in the UV−Vis−NIR region (350−1200 nm) of Ag nanoparticles by tannic-acid-aided reduction for 90 min in TiO_2_ nanoporous films by screen-printing (red line, thickness of 2400 nm after one-operation) and dip-coating (blue line, thickness of 600 nm after three operations)


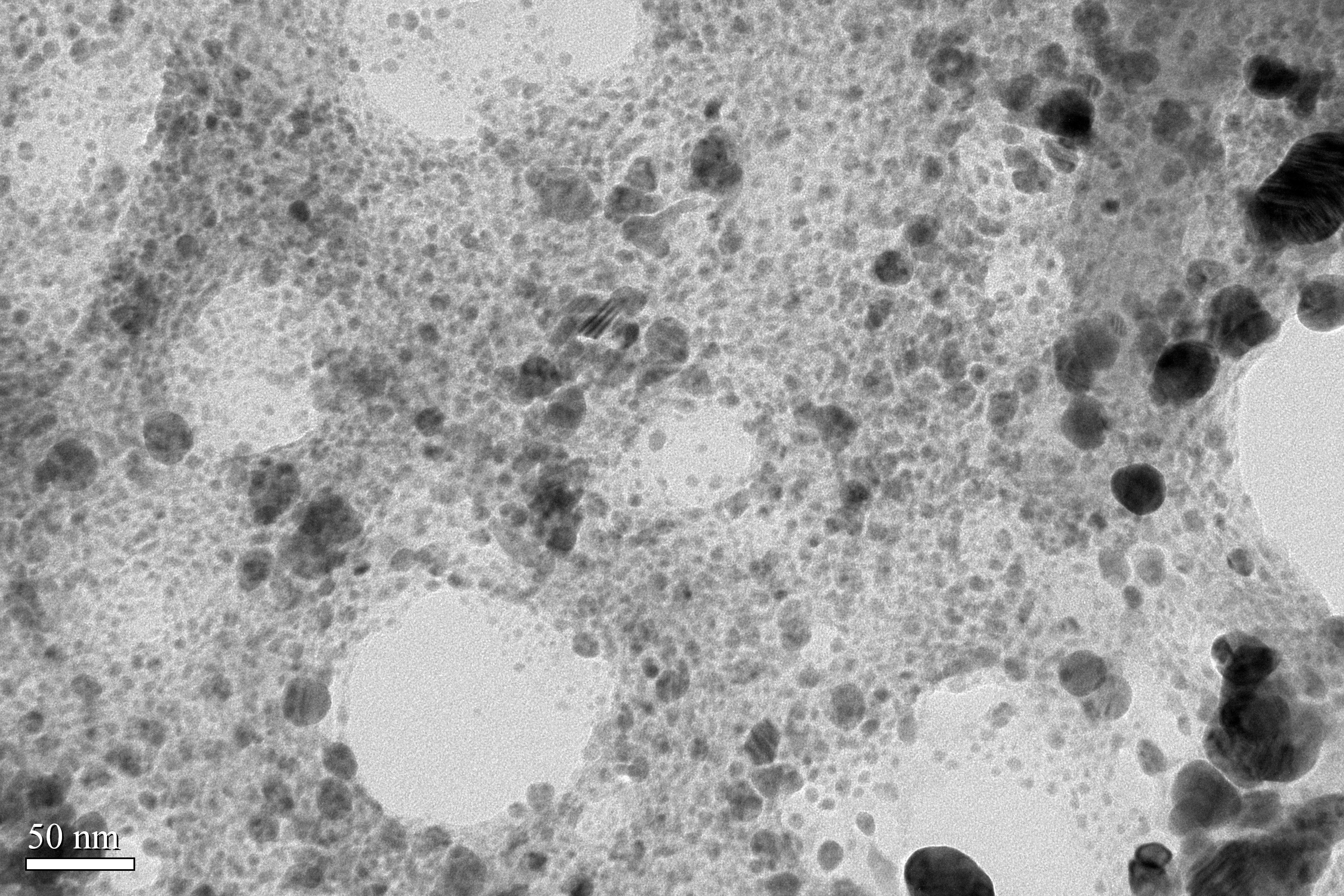


(a)

(b)

Figure S3 **(a)** TEM photographs of Ag NPs in the nano-composite film via UV-reduction for 20 min., TiO_2_ NPs in the films were dissolved with HF solution to remove interference. **(b)** The size distribution histograms and cumulative percentage of volume fraction of Ag NPs derived from TEM photographs.

365nm laser

Different film thickness

(365nm) power

density detector

(a)


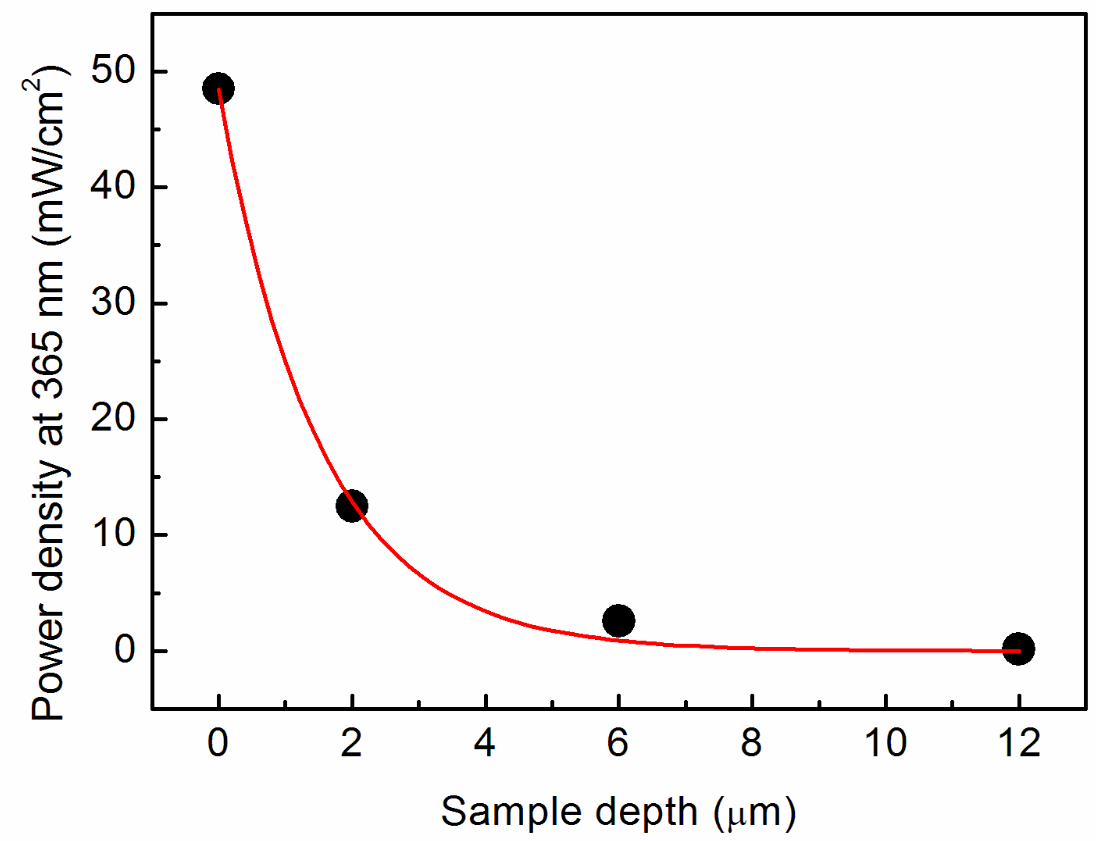


(b)

Figure S4 (**a)** Optical setup for measuring transmittance of TiO_2_ films with different thickness; (**b)** Transmitted power density at 365 nm of TiO_2_ films versus layer depth.


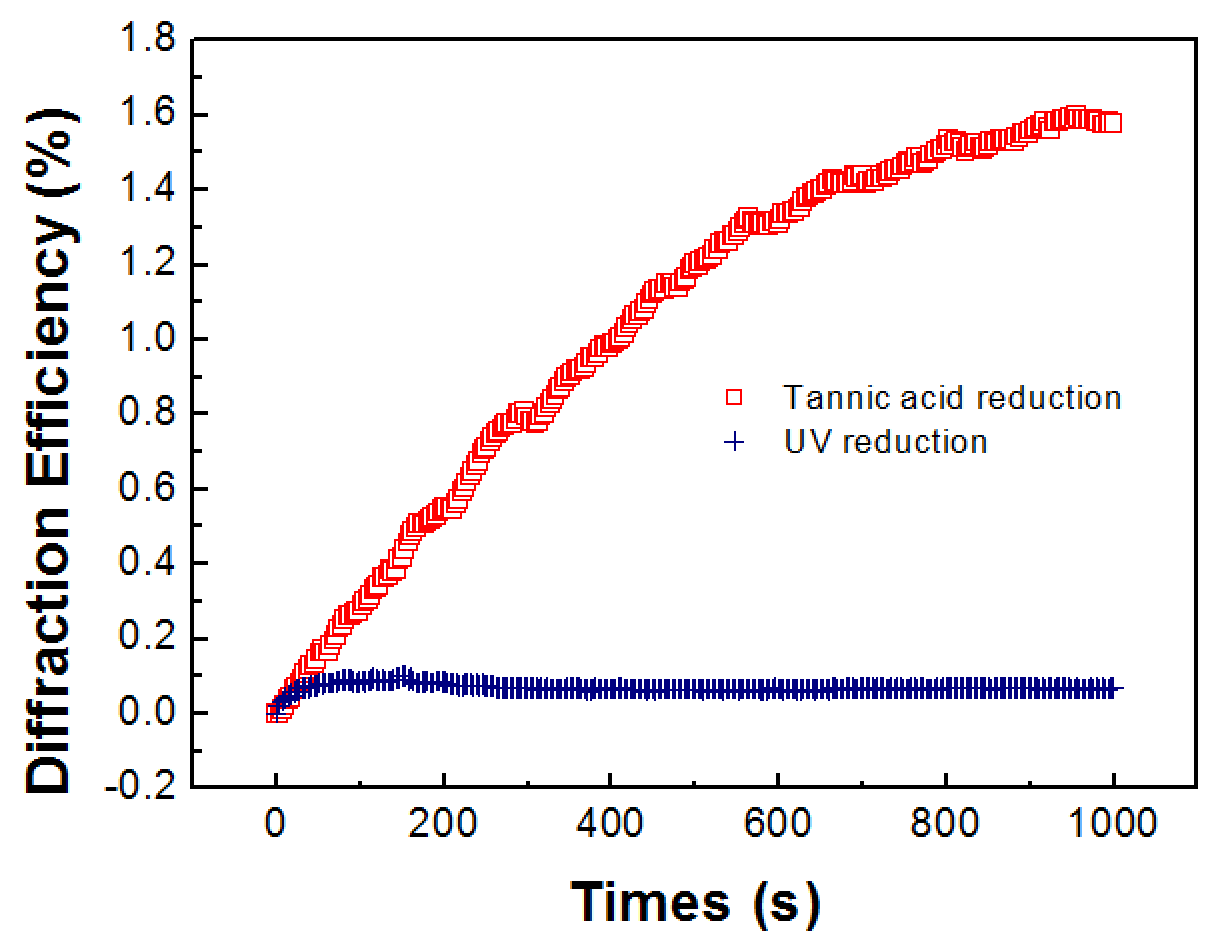


Figure S5 First-order diffraction efficiency versus time in (*ss*) recording configuration in the Ag/TiO_2_ nanocomposite films fabricated by tannic acid and UV reduction.
